# The first complete mitochondrial genome of eggplant (*Solanum melongena*)

**DOI:** 10.1080/23802359.2021.1878948

**Published:** 2021-03-01

**Authors:** Dandan Li, Weiliu Li, Guiyun Gan, Wenjia Li, Yaqin Jiang, Xuyu Liang, Riyuan Chen, Yikui Wang

**Affiliations:** a Institute of Vegetable Research, Guangxi Academy of Agricultural Sciences, Nanning, China

**Keywords:** *Solanum*, mitochondrial genome, RNA editing, phylogenetic relationships

## Abstract

Eggplant is an important vegetable crop because of its rich nutrition, but to date no mitochondrial genome has been reported. In this study, the complete mitochondrial genome of the eggplant was sequenced. The complete mitochondrial genome was 498,136bp, linear structure, containing 54 protein-coding genes, four rRNAs, and 32 tRNAs. The phylogenetic tree supported the hypothesis that the eggplant is most closely related to *Solanum tuberosum* and *Solanum lycopersicum*.

*Solanum* is one of the largest and the most economically important family in the *Solanaceae*, which encompasses more than 1500 species (Bohs and Olmstead, 1997) and is widely distributed in the tropical and subtropical regions of the world (Whalen 1984; Levin et al. 2006; Dupin et al. 2017). Eggplant is an important *Solanaceae* crop; however, when compared with other *Solanaceae* plants (such as tobacco, potato, tomato and pepper), the research work of eggplant is relatively backward. Now, we reported the complete mitochondrial genome of *Solanum melongena*, which is based on the next-generation sequence. We believe that our study provides the fundamental information for unraveling the evolution and domestication of the eggplant and may ultimately lead to further improvement of *Solanaceae* crops.

Here, we present the complete mitochondrial genome of eggplant collected from the Vegetable Research Institute, Guangxi Academy of Agricultural Science (28°N and 118°E), Guangxi Province, China. The identification was confirmed by YiKui Wang. The material was deposited at the Seed Bank of Guangxi Academy of Agricultural Science (accession number: 177).

## DNA extraction, genome sequencing, assembly, and genome analysis

Approximately 5 g of fresh leaves was harvested for mtDNA isolation using an improved extraction method (Chen et al. 2011). After DNA isolation, 1 μg of purified DNA was fragmented to construct short-insert libraries (insert size 430 bp) according to Illumina’s instructions, then sequenced on the Illumina Hiseq 4000 (Erik et al. 2011). The high molecular weight DNA was purified and used for PacBio library preparation, Blue Pippin size selection, then sequenced on the Sequel Sequencer. Prior to assembly, Illumina raw reads were filtered firstly. This filtering step was performed in order to remove the reads with adaptors, the reads showing a quality score below 20 (**Q **<** **20), the reads containing a percentage of uncalled based (‘N’ characters) equal or greater than 10% and the duplicated sequences. The mitochondrial genome was reconstructed using a combination of Pacbio Sequel and the Illumina Hiseq data, and the following three steps were used to assemble mitochondria genomes. First, Assemble the genome framework by the both Illumina and Pacbio data using SPAdesv3.10.1 (Antipov et al., 2016). Secondly, verifying the assembly and completing the circle or linear characteristic of the mitochondria genome, filling gaps if there were. Third, clean reads were mapped to the assembled mitochondria genome to correct the wrong bases, judge if there is any insertion and deletion.

The mitochondrial genes were annotated using homology alignments and *de novo* prediction, and the Evidence Modelerv1.1.1 was used to integrate the gene set (Haas et al. 2008). Transfer RNA (tRNA) genes and ribosome RNA (rRNA) genes were predicted by tRNA scan-SE (Lowe and Eddy 1997) and rRNA mmer 1.2 (Lagesen et al. 2007). Then, Blast (Altschul et al. 1990) search (E-value ≤ 1e–5, minimal alignment length percentage ≥ 40%) gene sequence against 5 databases for gene function annotation was used. The five databases are: KEGG (Knehisa 1997; Kanehisa et al. 2004, 2006) (Kyoto Encyclopedia of Genes and Genomes), COG (Tatusov et al. 1997, 2003) (Clusters of Orthologous Groups), NR (Non-Redundant Protein Database databases), Swiss-Prot (Magrane 2011), and GO (Ashburner et al. 2000) (Gene Ontology).

The complete mitochondrial genomes of other *Solanaceae* plants were downloaded from NCBI. ClustalW was used to align the mtDNA sequences under default parameters (Larkin et al. 2007), and the alignment was checked manually. The maximum-likelihood (ML) methods were performed for the genome-wide phylogenetic analyses using PhyML3.0 (Guindon et al. 2010). Nucleotide substitution model selection was estimated with j Model Test 2.1.10 (Darriba et al. 2012) and Smart Model Selection in PhyML 3.0. The model GTR + G was selected for ML analyses with 1000 bootstrap replicates to calculate the bootstrap values (BS) of the topology. The results were treated with iTOL 3.4.3 (Letunic and Bork 2016).

## Conclusions

**Figure 1.**
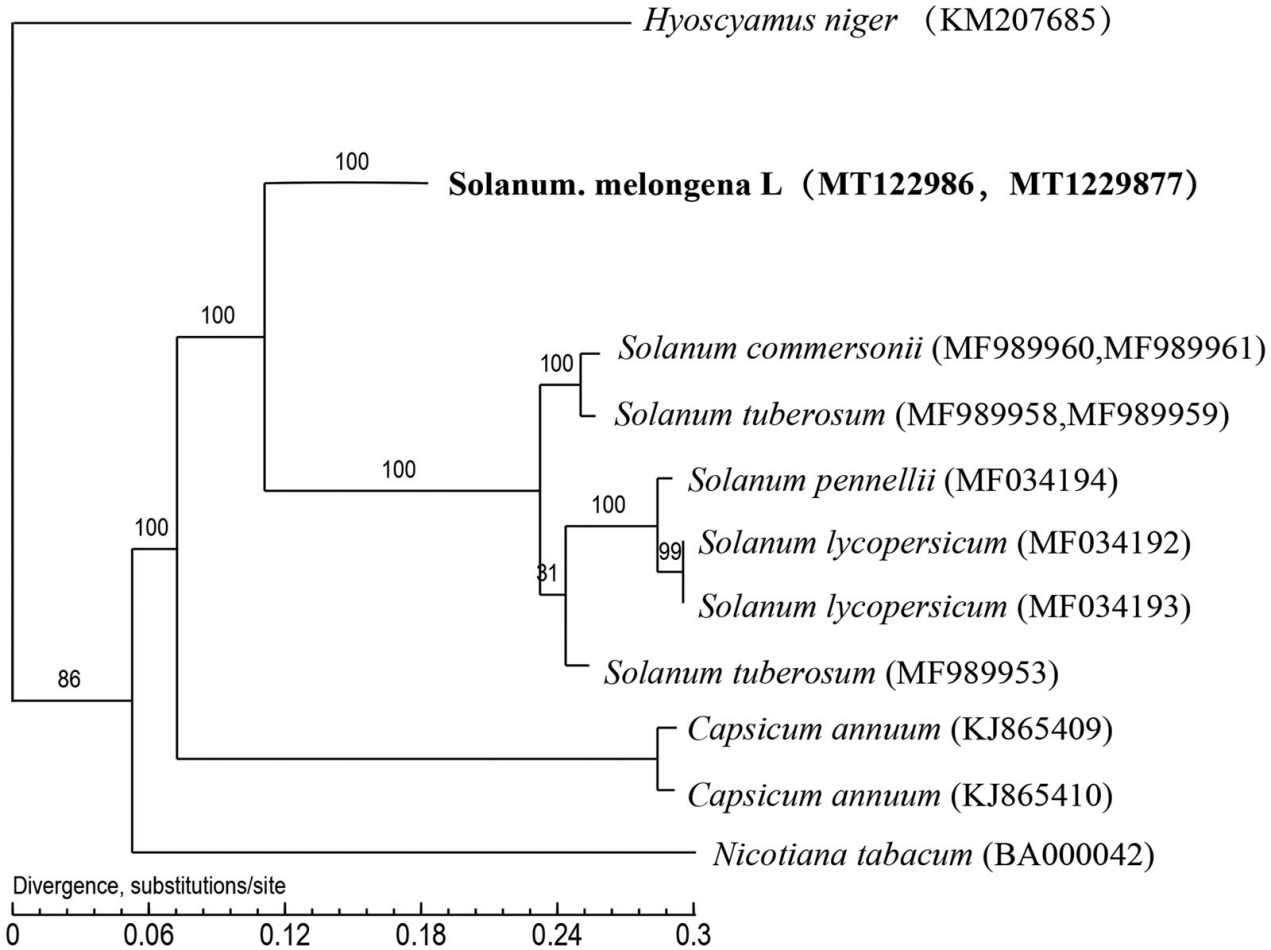
Evolutionary tree analysis of Solanaceae crops.

The complete mitochondrial genome was 498,136 bp (linear structure, two congtigs) and the GC content was 43.9%. There were 54 protein genes, 32 tRNAs and 4 rRNAs annotated. The percentage of three type of gene length is 8.28%, 1.01% and 0.49%, respectively. Through data analysis, we also found 264 SSRs and 285 edit sites. From the constructed phylogenetic tree, we use complete mitogenome sequences (Figure 1). The phylogeny tree supported the assertion that the eggplant is most closely related to *Solanum tuberosum* and *Solanum lycopersicum*.

## Data Availability

The genome sequence data that support the findings of this study are openly available in GenBank of NCBI at (https://www.ncbi.nlm.nih.gov/) under the accession MT122986 and MT122987. The associated SRA number is SAMN16746491.
